# Assessment of faecal calprotectin in neonatal calf diarrhoea using a human ELISA kit: Associations with faecal score and serum biochemical markers

**DOI:** 10.1002/vro2.70044

**Published:** 2026-07-15

**Authors:** Narges Lotfalizadeh, Farima Jahromi, Alborz Yadollahi, Saeed Nazifi

**Affiliations:** ^1^ Department of Clinical Sciences School of Veterinary Medicine Shiraz University Shiraz Iran

**Keywords:** AGR, calprotectin, diarrhoea, faecal biomarkers, neonatal calf diarrhoea

## Abstract

**Background:**

Diarrhoea in newborn calves, which occurs in the first month of life and is caused by various pathogens, poses a significant threat to livestock industries. Therefore, timely diagnosis of enteritis in calves can be advantageous.

**Objective:**

This study aimed to determine the efficacy of faecal calprotectin as a biomarker indicating gastrointestinal inflammation and a method to distinguish diarrhoeic calves from healthy calves. Additionally, the applicability of a human enzyme‐linked immunosorbent assay (ELISA) kit for measuring faecal calprotectin in bovine samples was evaluated. The relationship between calprotectin and faecal score as an index of diarrhoea severity, and the correlation between calprotectin levels, albumin, albumin‐to‐globulin ratio (AGR) and serum electrolytes has also been investigated.

**Methods:**

This study enrolled 34 Holstein neonatal calves, including 12 with diarrhoea and 22 healthy controls. A clinical assessment of faecal consistency was performed, and faecal samples were collected for ELISA measurement of calprotectin. Blood samples were taken to determine serum albumin and globulin concentrations, from which the AGR was calculated. The serum sodium (Na) and potassium (K) concentrations were also measured. Using SPSS (version 21) and appropriate statistical tests, associations between faecal calprotectin, albumin, AGR, Na, K and faecal scores were investigated.

**Results:**

Compared with healthy calves, diarrhoeic calves had higher calprotectin levels and lower AGR levels. The serum K levels in diarrhoeic calves were higher, while the serum Na levels were lower. Faecal calprotectin concentration and serum Na levels were negatively correlated. Calves with faecal score 3, which indicated severe watery diarrhoea, had significantly higher calprotectin levels than those with lower scores.

**Conclusion:**

Faecal calprotectin could represent a potential inflammatory biomarker for the diagnosis of neonatal calf diarrhoea, along with serum indicators.

## INTRODUCTION

Neonatal calf diarrhoea, which can be caused by a range of factors, including bacterial, viral and non‐infectious agents, remains the leading cause of illness and mortality among dairy calves worldwide before weaning.^1^ The four main gastrointestinal pathogens that cause diarrhoea in newborn dairy calves are *Cryptosporidium parvum*, rotavirus, coronavirus and *Escherichia coli*.^2^ Diarrhoeic calves, as well as those that have recovered from diarrhoea, spread pathogenic organisms into their environment and are considered primary sources of contamination.^3^ Consequently, prompt diagnosis and efficient treatment are necessary for managing calf diarrhoea.^4^ Due to the lack of appropriate diagnostic procedures, it is challenging to determine whether the symptoms observed in diarrhoeic calves are the primary cause of the condition or are secondary effects of the pathological process.^5^, ^6^

**FIGURE 1:**
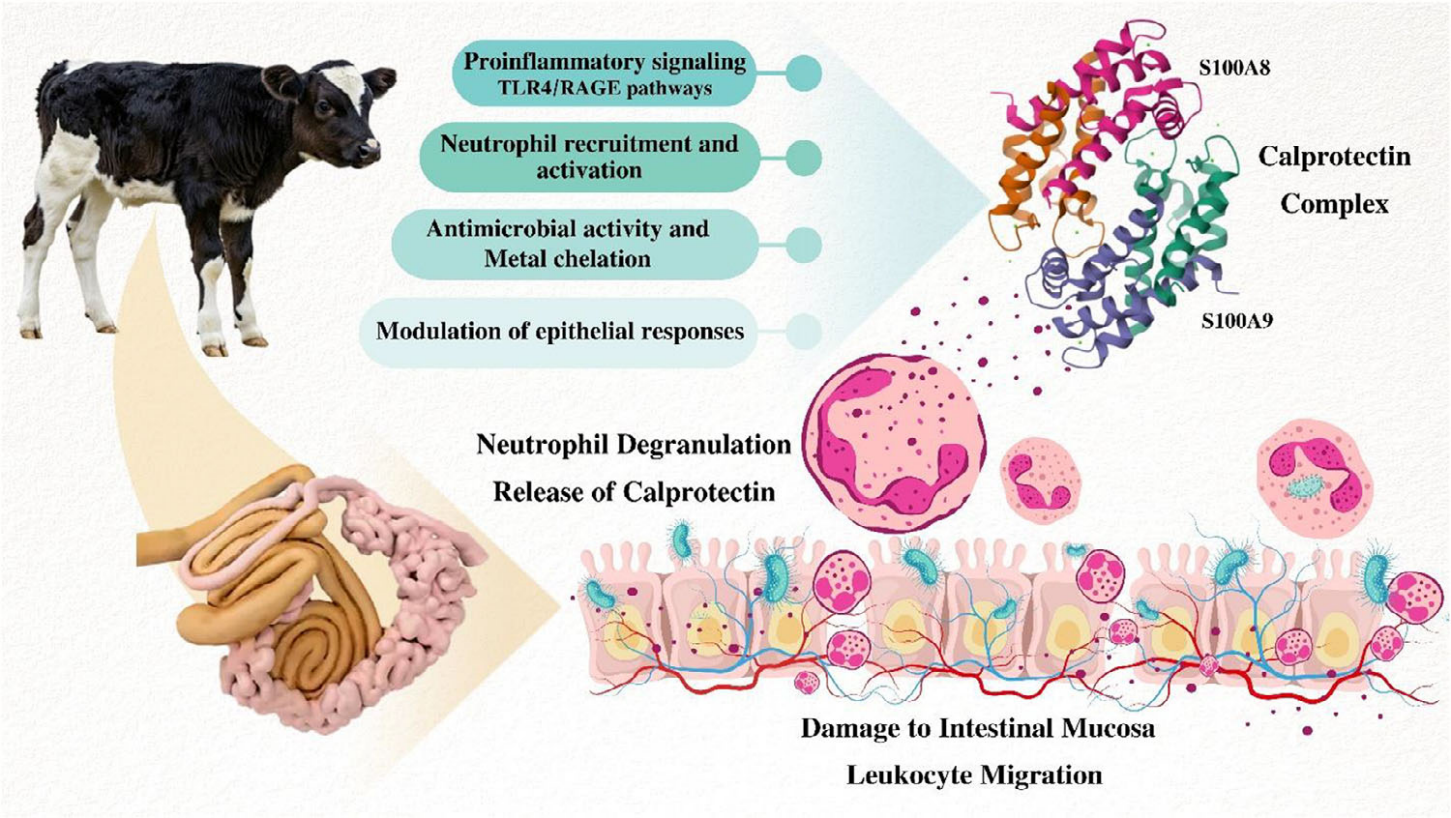
Schematic representation of calprotectin presence in the intestines. During the process of neutrophil margination and migration to the area of the damaged intestine, calprotectin is released. Calprotectin promotes the recruitment and activation of neutrophils through chemotaxis and adhesion, leading to a local inflammatory response. Antimicrobial activity is exerted on the intestinal lumen through zinc and manganese chelation. Signalling pathways such as Toll‐like receptor 4 (TLR4) and the receptor for advanced glycation end products (RAGE) are activated, resulting in an increase in pro‐inflammatory cytokines. Consequently, it causes dysregulation of the epithelium barrier and mucosal injury, perpetuating inflammation within the small intestine. This figure was created by the authors.

Calprotectin is a calcium‐binding protein from the S100 family.^7^ Neutrophils produce this substance primarily, while macrophages and monocytes produce lesser amounts.^8^ Inflammation determines the amount of calprotectin present in various body fluids, including faeces, which have concentrations that are six times those in plasma.^9^ It is useful to measure faecal calprotectin as a surrogate marker of gastrointestinal inflammation.^7^ The heterodimeric calprotectin complex, composed of S100A8 and S100A9, contributes to many intracellular and extracellular processes.^10^, ^11^, ^12^ Moreover, calprotectin acts as a damage‐associated molecular pattern and binds to pattern recognition receptors such as Toll‐like receptor 4 and the receptor for advanced glycation end products, thereby initiating or augmenting inflammation, including neutrophil recruitment and endothelial adhesion^10^, ^13^, ^14^, ^15^ (Figure 1).

The pathologic process of diarrhoea in calves results in changes in serum biochemistry, including alterations in electrolytes and proteins.^16^ Diarrhoea may result in dehydration, electrolyte imbalances, metabolic acidosis and hypovolaemia, regardless of its cause.^17^ Recent studies found that diarrhoeic calves’ serum Na levels were significantly lower than those of healthy calves, while their serum K levels were significantly higher, suggesting that electrolyte concentrations are associated with the occurrence of metabolic acidosis and the severity of diarrhoea in calves.^17^, ^18^, ^19^

Serum albumin‐to‐globulin ratio (AGR) is calculated by dividing serum albumin (Alb) by serum globulin.^20^ Albumin can be used to assess inflammation and nutritional status.^21^, ^22^ Globulins include immunoglobulins and acute phase proteins, such as haptoglobin, fibrinogen, serum amyloid A (SAA) and C‐reactive protein.^23^ There is a close connection between the levels of globulin and the immune and inflammatory systems of individuals.^24^ Evidence suggests that serum AGR has a reliable predictive value for inflammatory conditions.^25^ A negative correlation was observed between systemic inflammation and the AGR measure.^24^

Faecal calprotectin has been well established in human medicine as a non‐invasive marker of intestinal inflammation,^26^ but its applicability and diagnostic use in calves with diarrhoea are unclear. Faecal calprotectin analysis could be a practical and simple tool for neonatal calves on farms. Additionally, evaluating its relationship with systemic indicators such as serum Alb concentration and the AGR may provide insight into the causal relationship between diarrhoea and systemic protein changes.

This study aimed to compare faecal calprotectin levels between diarrhoeic and healthy calves and to determine whether faecal calprotectin could be used as a diagnostic marker, and to explore the applicability of a human faecal calprotectin enzyme‐linked immunosorbent assay (ELISA) kit for bovine faecal samples. Correlations of faecal calprotectin levels with Alb, a negative acute phase protein, and AGR were evaluated to determine its potential as a systemic inflammation biomarker. Additionally, this study assessed the association between faecal calprotectin and serum electrolytes, including Na and K. As an indicator of the condition's severity, the relationship between faecal score and calprotectin concentrations was analysed.

## MATERIALS AND METHODS

### Study design and animals

This study was designed as an observational case–control study to compare faecal calprotectin concentrations between diarrhoeic and healthy calves and to determine the association between serum protein parameters and faecal calprotectin concentrations.

A total of 34 Holstein calves aged between 3 and 21 days from a commercial dairy farm with similar feeding conditions were included in this study. Calves less than 3 days old were excluded from the study because colostrum and meconium proteins could interfere with the test. At the time of sampling, none of the selected calves exhibited severe systemic illness, marked depression or advanced dehydration. Samples were collected during the early clinical stage of diarrhoea prior to therapeutic intervention under routine field management conditions. Furthermore, the selected calves had no congenital anomalies or a history of systemic disease, and they were not treated with antibiotics or anti‐inflammatory medications. Then, the calves were divided into two groups based on clinical signs and faecal consistency: healthy and diarrhoeic. Among the population, 22 calves had normal faecal consistency and were considered healthy, but 12 had loose to watery faeces and were classified as diarrhoeic. The diarrhoeic calves had a median age of 14 (range 5‒18) days, whereas the healthy calves had a median age of 15.5 (range 4‒21) days, with no apparent difference in age distribution between groups.

### Faecal scoring and diarrhoea definition

During sampling, faecal consistency was assessed using a standardised scoring system.^27^ Based on this system, 0 indicates normal, well‐formed faeces; 1 indicates soft faeces; 2 indicates loose faeces; and 3 indicates watery diarrhoea. A calf with a faecal score of 2 or higher was classified as diarrhoeic, while one with a faecal score of 0 or 1 was considered healthy.

### Sample collection and handling

Fresh faecal samples were collected directly from calves during spontaneous defecation into sterile containers and immediately stored at ‒20°C until analysis. Blood samples were collected by jugular venipuncture into plain tubes, allowed to clot at room temperature, and serum was isolated by centrifugation at 3000 rpm for 5 min. Following the separation of the serum, it was transferred into sealed microtubes and stored frozen at ‒20°C. The faecal samples as well as the serum samples were kept frozen for approximately 1 week. After samples were completely thawed at room temperature, they were subjected to analysis. In order to minimise potential degradation of analytes, no repeated freeze‒thaw cycles were performed.

### Faecal sample preparation

IDK Calprotectin stool ELISA kit (Immundiagnostik AG) instructions were followed to prepare faecal samples for ELISA analysis.^28^ Fifteen milligrams of the thawed faeces was applied using the stool sample application system (SAS). In the SAS tube, 1.5 mL of extraction buffer provided by the kit was prefilled, which provided a diluted calprotectin extraction solution. The tube was then vortexed until a homogeneous suspension was achieved. Following approximately 10 min of standing time to allow sedimentation, the clarified faecal extract was analysed.

### Calprotectin measurement

The IDK Calprotectin stool ELISA kit was used to determine faecal calprotectin concentrations. The standards, controls and diluted samples were placed in microplate wells coated with anti‐calprotectin antibodies. Incubation at room temperature was followed by five washes with the provided wash buffer to remove unbound proteins. After adding an enzyme‐conjugated secondary antibody, the reaction was incubated and washed. The substrate solution was applied, allowed to develop for 20 min, and then stopped using the stop solution provided. Analyses of optical density were performed at 450 nm through the Convergys EL‐Reader 96X microplate reader. The assay calibration curve was generated using five standards, covering a range of 0‒840 ng/mL, and concentrations of faecal calprotectin in samples were calculated accordingly. The assay was run with both high‐ and low‐concentration control samples of the kit to ensure quality control. Duplicate analyses were performed on all samples. Calprotectin concentrations measured by ELISA in the extract were converted from ng/mL of extract to ng/mL of faeces by accounting for the weight of the faecal sample and the volume of extraction buffer. Since the manufacturer does not provide detailed information regarding the exact dilution and conversion procedure used to derive standard concentration units, the results are reported in ng/mL according to the original assay specifications, allowing for easier comparison between samples.^28^

### Evaluation of bovine sample parallelism with kit control

To determine the reliability of the human IDK Calprotectin faecal ELISA kit in measuring calprotectin in bovine samples, a dilution series (1:2, 1:4, 1:8 and 1:16) was performed using the bovine sample and one of the kit controls (CTRL2). The measured concentration values were multiplied by the dilution factor, and the adjusted concentration was calculated to eliminate the dilution effect and evaluate the linearity of the kit response.^29^

### Serum measurements

The separated serum was used for the measurement of Na and K concentrations using the flame photometer (Fater Electronic Rizpardaz). The results were expressed in mmol/L. The concentrations of total protein (TP) and Alb in serum were measured using an Alpha Classic AT Plus autoanalyser (TS Technology; Sanjesh Co.). Measurements were expressed in g/dL. Albumin was subtracted from TP to calculate serum globulin concentration (globulin = TP ‒ Alb), and AGR was calculated by dividing Alb by globulin.

### Statistical analysis

The statistical analyses were conducted using SPSS (version 21). A Shapiro‒Wilk test was used to evaluate the normality of the data distribution. Because faecal calprotectin concentrations were not normally distributed, non‐parametric tests were used for analyses involving this variable. In contrast, parametric tests were applied to normally distributed variables such as serum TP, Alb, AGR, Na and K. Inflammatory biomarkers such as faecal calprotectin often exhibit positively skewed (non‐normal) distributions with substantial variability and occasional extreme values, a pattern common among markers of inflammation and observed in many clinical studies.^30^

Mann‒Whitney *U* tests were conducted to compare faecal calprotectin concentrations between diarrhoeic and healthy calves. At the same time, independent sample *T*‐tests were used to compare serum TP, Alb, AGR, Na and K. Spearman's rank correlation was employed to evaluate the association between faecal calprotectin concentrations and AGR, Alb, Na and K. The relationship between faecal consistency scores (0–3) and calprotectin concentrations was assessed using the Kruskal‒Wallis test. Receiver operating characteristic (ROC) curve analysis was performed to identify a preliminary cut‐off value for distinguishing between diarrhoeic and healthy calves. Results are reported with a 95% confidence interval, and *p*‐values less than 0.05 are considered statistically significant.

## RESULTS

### Cross‐species validation

**FIGURE 2:**
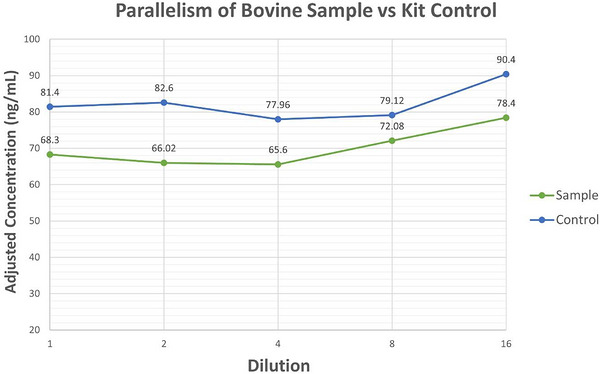
In a series of dilutions, bovine sample and kit control show a parallel pattern. Dilution series for a bovine sample (initial concentration = 68.30 ng/mL) and the kit control (initial concentration = 81.40 ng/mL) were prepared and measured using the kit. Dilution factors were multiplied by measured concentrations to calculate adjusted concentrations. To visualise parallelism, both series were plotted on a log_2_ *X*‐axis.

The scatter plot showed that the adjusted values of the bovine sample and the kit control were plotted with the *X*‐axis corresponding to dilutions and the *Y*‐axis corresponding to adjusted concentrations. The sample and control lines were almost horizontal, with the points corresponding to each dilution marked on the lines. The human ELISA kit was capable of detecting calprotectin in bovine faecal samples with acceptable dilution linearity and parallelism, supporting its possible applicability in this matrix (Figure 2).

### Comparison of faecal calprotectin and serum parameters between groups

There were marked differences in biochemical and faecal biomarker patterns between healthy and diarrhoeic neonatal calves in this study. Calprotectin concentrations in diarrhoeic calves were significantly higher than those in healthy calves (*p* = 0.009). Additionally, the AGR was significantly lower in diarrhoeic calves compared to healthy calves (*p* = 0.033). The comparison of serum TP and serum Alb concentrations in diarrhoeic and healthy calves did not show any statistically significant differences. In terms of serum electrolyte levels, the comparison indicated that diarrhoeic calves had significantly lower serum Na levels (*p* < 0.001) and higher serum K levels (*p* = 0.003) than healthy calves (Table 1).

**TABLE 1:** Comparison of faecal calprotectin (median (*Q*1‒*Q*3)) and serum parameters (mean ± SD) in healthy and diarrhoeic calves.

Parameter	Healthy	Diarrheic	*p*‐Value
Faecal calprotectin (ng/mL)	26.00 (19.5–32.73)	49.124 (31.56–221.09)	0.009
Total protein (g/dL)	5.51 ± 0.52	5.46 ± 0.59	0.396
Albumin (g/dL)	2.35 ± 0.15	2.20 ± 0.27	0.098
AGR	0.76 ± 0.11	0.70 ± 0.19	0.033
Na (mmol/L)	127.18 ± 4.13	115.75 ± 6.28	<0.001
K (mmol/L)	4.01 ± 0.46	4.58 ± 0.48	0.003

Abbreviation: AGR, albumin‐to‐globulin ratio.

### Correlations between faecal calprotectin and serum parameters

Spearman's correlation analysis demonstrated no significant association between faecal calprotectin and AGR (*ρ* = −0.023, *p* = 0.896). Albumin was not significantly correlated with faecal calprotectin either (*ρ* = 0.015, *p* = 0.932). Based on these findings, neonatal calves show individual differences in faecal calprotectin levels and systemic protein alterations. There was a statistically significant moderate negative correlation between faecal calprotectin concentration and serum Na levels (*ρ* = −0.425, *p* = 0.012). However, the correlation between serum K levels and faecal calprotectin did not reach statistical significance (*ρ* = 0.222, *p* = 0.207).

### Association between faecal score and calprotectin concentration

Among the 34 calves evaluated, 14 scored 0 in faeces, eight scored 1, eight scored 2, and four scored 3. There was a significant difference in the concentration of calprotectin between faecal score categories (*p* = 0.009) (Table 2). The differences between the groups were identified via post hoc pairwise comparisons using Dunn's tests. Calves with severe and watery diarrhoea (faecal score 3) had significantly higher calprotectin concentrations as compared to calves with lower faecal scores (*p* < 0.05).

**TABLE 2:** Distribution of calves' faecal scores and calprotectin concentrations.

Faecal score	Number of calves, *n*	Calprotectin (ng/mL), median (*Q*1–*Q*3)
0 (normal, well‐formed faeces)	14	26.00 (18.69–30.67)
1 (soft faeces)	8	26.47 (19.66–35.00)
2 (loose faeces, moderate diarrhoea)	8	30.04 (22.59–50.56)
3 (watery diarrhoea)	4	406.37 (141.27–904.81)

*Note*: The faecal scoring system was adapted from Renaud et al.^27^

### Diagnostic performance of faecal calprotectin

The area under the curve (AUC = 0.77) indicates an acceptable discriminative capacity of faecal calprotectin for detecting neonatal calf diarrhoea. Considering the analysis and observed distribution of calprotectin values, a cut‐off of 30.625 ng/mL was selected for a clinically meaningful threshold. Using this threshold, diarrhoeic calves are distinguished from healthy calves with a sensitivity of 83.3% and a specificity of 68.2%.

## DISCUSSION

The diagnosis of gastrointestinal inflammation in cattle has been made using several faecal markers. Some of them include mucosal antibodies immunoglobulin A and immunoglobulin G, faecal lactoferrin and faecal calprotectin.^31^, ^32^ A recent comparative study revealed a direct correlation between faecal lactoferrin and calprotectin levels. Calprotectin was a better indicator of bovine enteritis than lactoferrin, as it was not affected by faecal water content.^31^ In veterinary medicine, calprotectin has been studied and discussed in the serum, faeces, urine and even saliva of domestic animals.^29^, ^33^, ^34^, ^35^, ^36^ The use of human kits in many animals and their reliable results indicate a promising level of similarity in the calprotectin protein subunits, especially the S100A8 subunit, between humans and some animals.^37^ A commercially available human faecal calprotectin ELISA kit was used in the current study due to the limited accessibility of bovine calprotectin assays within our setting. Despite having been developed for use in human samples, it has been demonstrated in prior veterinary research that human‐derived calprotectin tests could be used on bovine faecal samples after undergoing analytical validation.^31^, ^37^, ^38^ In this study, dilution linearity was evaluated to assess analytical performance in bovine faecal extracts, demonstrating acceptable analytical performance. Nevertheless, further species‐specific validation studies, including assessment of recovery, repeatability and detection limits in bovine matrix, are still required before routine clinical application can be fully recommended.

This study showed that faecal calprotectin in calves can be a useful indicator for identifying neonatal calf diarrhoea. Higher calprotectin levels in diarrhoeic calves demonstrate local inflammation of the gastrointestinal tract and changes independently of the level of serum AGR as an indicator of systemic inflammation. Moreover, faecal calprotectin levels increased with increasing diarrhoea score, suggesting a potential association with disease severity. Calprotectin accumulates in certain barrier tissues, particularly the intestinal mucosa, where persistent immune activation occurs.^11^, ^39^ In these tissues, neutrophils and other myeloid cells are continuously recruited and activated, leading to the sustained local release of calprotectin to mucosal tissues and the lumen.^8^, ^40^ Calprotectin is actively involved in the inflammatory processes by instigating immune responses within tissues and by antimicrobial activity through the removal of certain divalent metal ions that are essential for microbial survival.^41^ Elevated calprotectin levels indicate ongoing immune activity and barrier dysfunction rather than merely transient tissue damage.^42^ This also applies to calves with diarrhoea secondary to infectious enteritis, since the loss of their epithelial barrier is caused by intense and prolonged neutrophil infiltration into the mucosal tissues and the consequent persistent secretion of calprotectin into the intestinal lumen.^43^, ^44^

In another research on faecal calprotectin in diarrhoeic calves, no significant correlation was found between calprotectin levels in serum TP and globulins.^44^ The present study showed a similar finding, with faecal calprotectin increasing as an indicator of local intestinal inflammation, independently of serum inflammatory markers. Furthermore, in their study, there was a moderate negative correlation between faecal calprotectin and body temperature and a moderate positive correlation with capillary refill time and lactate levels in terms of clinical symptoms.^44^ Considering metabolic lactic acidosis occurs in more severe cases during diarrhoea, and diarrhoeic calves develop hypothermia under more serious conditions, these findings are in line with this study. High faecal scores can also be associated with more severe clinical symptoms and dehydration due to watery diarrhoea, as well as a higher faecal calprotectin concentration. In this study, faecal scoring was used to categorise diarrhoea by clinical faecal consistency, while acknowledging that this classification pertains to clinical severity rather than the underlying cause.

The lack of correlation between faecal calprotectin and both serum Alb and AGR can be understood on the basis of different biological roles and kinetics of these variables. Calprotectin is a locally derived biomarker, released mainly from activated neutrophils and therefore represents a rapid and site‐specific intestinal inflammatory response.^8^, ^45^ In contrast, Alb as a negative acute phase protein exhibits slower and more delayed changes in systemic inflammatory conditions.^46^ Albumin levels are additionally influenced by hydration status, nutritional balance and hepatic synthesis.^46^, ^47^ Differences in biological origin, systemic versus local response and temporal dynamics may account for the absence of a significant association between faecal calprotectin and these serum biomarkers.

The presence of hyponatraemia and hyperkalaemia is common in neonatal calf diarrhoea.^48^ The current study demonstrated that faecal calprotectin concentrations were negatively correlated with serum sodium concentrations in diarrhoeic calves, indicating that intestinal inflammation leads to sodium depletion. Dehydration and impaired gastrointestinal absorption cause hyponatremia in neonatal diarrhoea.^19^, ^49^ In contrast, serum potassium levels were not significantly associated with faecal calprotectin. In diarrhoeic calves, potassium homeostasis is influenced by multiple mechanisms, including faecal potassium loss, metabolic acidosis‐induced transcellular shifts and prerenal azotemia due to dehydration.^50^, ^51^ These concurrent processes may mask the direct relationship between enteritis and serum potassium levels.

Measurements of acute phase proteins such as SAA along with serum calprotectin have been carried out to distinguish causes of diarrhoea in recent studies.^52^ When coronavirus‐induced diarrhoea was compared with *E. coli*‐induced diarrhoea, serum calprotectin and SAA levels were reported higher.^52^ A study showed that faecal calprotectin is more than six times as accurate as serum calprotectin for diagnosing acute infectious diarrhoea in newborn calves, suggesting that measuring faecal calprotectin can be as useful as SAA.^53^ Therefore, measuring calprotectin in faeces is better than serum and can also be considered on farms due to easier sampling. Also, the pattern of changes in faecal calprotectin differs between diarrhoea caused by *E. coli* and that caused by viral agents, and this pattern can help differentiate the aetiology of diarrhoea.^53^ The main drawback of the current study is the lack of aetiological diagnosis of diarrhoea, which limits our ability to evaluate the impact of individual pathogens on faecal calprotectin. However, this experimental design corresponds to the actual practice in the veterinary field, where neonatal calf diarrhoea is commonly treated as a clinical syndrome based on calf age, faecal scoring and clinical presentation, rather than routinely confirming the responsible pathogens due to constraints on cost, time and the unavailability of precise diagnostic testing.^54^, ^55^

Faecal calprotectin may complement conventional serum biomarkers, such as acute phase proteins, in the assessment of gastrointestinal inflammation, as it reflects inflammatory processes occurring within the intestinal tract.^53^ It would be advantageous for future research to evaluate and compare other systemic inflammation indices, such as haematological ratios such as neutrophil‐to‐lymphocyte ratio, platelet‐to‐lymphocyte ratio, systemic inflammation response index and systemic inflammation index, which have recently received much attention, alongside calprotectin concentrations in neonatal calf diarrhoea.^56^, ^57^, ^58^, ^59^

While faecal sampling is non‐invasive and convenient, sample's freshness and storage conditions can affect faecal index measurements. Therefore, it is critical to follow standard procedures for faecal sample collection and cold storage to ensure accurate calprotectin measurements. Faecal tests also have stricter hygiene considerations than serum tests, and there is a risk of microbial contamination spreading in the laboratory.^60^

In the present study, the analysis of diagnostic performance should be approached with suitable caution. The small sample size, especially within the diarrhoeic group, could influence the robustness and generalisability of the cut‐off. Although this ROC analysis and the proposed cut‐off are preliminary and not ideal, it can be helpful for distinguishing calves with a biological inflammatory process in the intestines and isolating those at higher risk of developing gastrointestinal diseases. Research involving a larger number of calves would be extremely valuable, as the results can be generalised to the entire population. Additionally, the pattern of changes in faecal indicators, such as calprotectin, could be assessed in relation to feeding, age and possible causes of inflammation. In this case, the reference range and cut‐offs for diagnosing affected animals can be determined more accurately. This study covers a relatively broad age range within the neonatal period; however, age‐related variations in intestinal and immune development could affect faecal calprotectin levels. Therefore, future research involving more age‐homogeneous groups could produce more precise results.

Currently, the use of faecal calprotectin has been investigated in some research and pilot studies.^31^, ^37^, ^44^, ^53^ Still, it has not yet reached a stage applicable in clinical practice, and it is not yet used on farms as a diagnostic tool. One of the drawbacks that limits the clinical utility of calprotectin is the time‐consuming nature of its measurement using existing ELISA methods, making it difficult and impractical to routinely perform in large livestock populations. In the future, a serological rapid kit could be designed to enable fast differentiation between calves experiencing diarrhoea and those at risk of developing diarrhoea. Efficient identification of these calves can prevent further transmission of infectious agents within the herd and enable prompt treatment. Establishing a routine testing system is important to detect diarrhoeic calves at early stages. Considering the present study's findings, along with the recent studies, faecal calprotectin can be considered as a as a complementary, potential biomarker of intestinal inflammation alongside conventional clinical parameters when it comes to the diagnosis of diarrhoea. In the future, prospective longitudinal studies are needed to determine whether faecal calprotectin can indicate early intestinal changes before clinical diarrhoea and to evaluate its potential role in tracking disease progression and associated clinical and biochemical parameters over time in calves.

## CONCLUSION

From a practical perspective, the human ELISA kit used in this study was able to differentiate between healthy and diarrhoeic calves, suggesting its potential as an accessible tool in field conditions after proper validation, particularly where bovine‐specific assays are limited. Faecal markers, such as calprotectin, and modified systemic markers, such as AGR, that have been corrected for the effect of dehydration, both provide valuable insights into neonatal calf diarrhoea. Higher levels of faecal calprotectin show potential for diagnosing neonatal calf diarrhoea when measured at the early stages, before severe clinical signs appear. However, faecal calprotectin, AGR and Alb do not correlate with each other. As faecal calprotectin levels are negatively correlated with serum sodium concentration, and since sodium is excreted from the body along with water in severe diarrhoea, high faecal calprotectin levels could indicate disease severity. The relationship between calprotectin and faecal score also supports this. Thus, faecal calprotectin measurement can also assist in staging diarrhoea and assessing severity. Future studies could also evaluate the applicability of faecal calprotectin as a biomarker in other ruminant species to determine its broader potential in veterinary medicine. However, further validation studies are required before broader application can be recommended.

## AUTHOR CONTRIBUTIONS

*Conceptualisation*: Saeed Nazifi and Narges Lotfalizadeh. *Methodology*: Saeed Nazifi, Narges Lotfalizadeh, Farima Jahromi and Alborz Yadollahi. *Formal analysis and investigation*: Saeed Nazifi, Narges Lotfalizadeh and Alborz Yadollahi. *Writing—original draft preparation*: Narges Lotfalizadeh and Saeed Nazifi. *Writing—review and editing*: Narges Lotfalizadeh, Saeed Nazifi and Farima Jahromi. *Supervision*: Saeed Nazifi. All the authors checked and approved the final version of the manuscript for publication in the present journal.

## CONFLICTS OF INTEREST

The authors declare they have no conflicts of interest.

## ETHICS STATEMENT

All applicable international, national and/or institutional guidelines were followed. This study was conducted under routine field conditions, and faecal sampling is a non‐invasive procedure. Blood sampling for serum analyses was performed in minimal volumes and under standard veterinary procedures with appropriate animal handling to ensure welfare and minimise stress.

## Data Availability

The datasets generated during and/or analysed during the current study are available from the corresponding author upon reasonable request.
